# Molecular sexing and population genetic inference using a sex-linked microsatellite marker in the nine-spined stickleback (*Pungitius pungitius*)

**DOI:** 10.1186/1756-0500-4-119

**Published:** 2011-04-12

**Authors:** Takahito Shikano, Gábor Herczeg, Juha Merilä

**Affiliations:** 1 Ecological Genetics Research Unit, Department of Biosciences, University of Helsinki, P.O. Box 65, FI-00014, Helsinki, Finland

## Abstract

**Background:**

Sex-specific DNA markers can serve as tools for molecular sex identification, as well as for population genetic inferences. We investigated the potential utility of a microsatellite marker located on sex chromosomes for molecular sexing of Fennoscandian nine-spined sticklebacks (*Pungitius pungitius*). In addition, we assessed the patterns of allelic differentiation between X and Y chromosomes across the populations to examine if the sex chromosomes had been highly differentiated prior to the postglacial recolonization of Fennoscandia.

**Findings:**

A clear and consistent sex difference in allele size distribution was observed at the Stn19 locus throughout the 15 populations investigated. Males were distinguishable by the presence of distinct male-specific alleles, which were lacking in all females. There was no indication of recombination between sex and the Stn19 locus in the 647 individuals tested. The degree of genetic differentiation between the X and Y chromosomes was much higher than that of interpopulation differentiation in the respective chromosomes.

**Conclusions:**

Our results indicate that the Stn19 locus can be used for molecular sex identification in Fennoscandian nine-spined sticklebacks. The consistent pattern of high allelic differentiation between the X and Y chromosomes in these populations suggests that the sex chromosomes were already highly differentiated prior to the postglacial recolonization of Fennoscandia.

## Background

Unlike mammalian or avian species, fish exhibit diverse sex-determination systems [1]. Even though many fish do not possess heteromorphic sex chromosomes, genetic sex determination has been found in a wide variety of fish species [1]. This was initially demonstrated in species displaying sex-linked phenotypic traits such as skin pigmentation or coloration [2-5]. Similarly, sex-linked allozyme markers have been identified in some fish species [e.g. [6-8]]. Nevertheless, it is important to note that sex-linked phenotypic traits or allozyme markers are not necessarily encoded by sex-linked genes, since the sex linkage can be a consequence of sex-specific gene expression of autosomal genes [1]. In contrast, DNA polymorphisms are not affected by altered physiological and environmental conditions, and thus are better suited for sex identification. DNA markers tightly associated with phenotypic sex have been reported in several fish species [9-13]. Such DNA markers can serve as simple and reliable diagnostic tools for sex identification of immature individuals.

Sex-linked DNA markers can also provide significant insights into demographic history and phylogenetic relationships among populations. In particular, polymorphic Y chromosome markers are useful in tracing paternal lineages in wild populations [14,15]. In humans, Y chromosome markers have been employed for the evaluation of the origin of non-African populations and sex-biased migration [16,17]. Despite their advantages, the utility of Y chromosome polymorphisms is limited to humans and some other mammals [15]. This is partly due to the difficulty of discovering polymorphic Y chromosome markers for organisms in which sex chromosomes are not highly differentiated [15]. As such, few studies have been conducted on phylogenetic and population genetic analyses using polymorphic Y chromosome markers in fish [18].

Sticklebacks (Gasterosteidae) are widely used as model organisms in evolutionary biology [19-21]. The nine-spined stickleback (*Pungitius pungitius*) is a cold-water adapted species having a circumpolar distribution in the Northern Hemisphere and is found in a wide variety of habitats [19]. This species possesses a heteromorphic XY chromosome pair corresponding to three-spined stickleback linkage group 12 [22,23]. Microsatellite analyses have revealed a high degree of genetic differentiation between the X and Y chromosomes over most of their length in nine-spined sticklebacks (T. Shikano, Y. Shimada & J. Merilä, unpublished). This implies that microsatellite markers can be used as a diagnostic tool for sex identification in this species. However, the practical utility of this approach needs to be validated as the results were obtained using only two particular populations. The necessity of such a validation is exemplified by previous studies that have demonstrated population-specificity of molecular sex identification [10,11,24].

We investigated the potential utility of a microsatellite marker located on sex chromosomes for molecular sex identification in a large number of Fennoscandian nine-spined stickleback populations. These populations are highly subdivided from each other as a result of their postglacial colonizations following the last glacial maximum (ca. 12 000 years ago) [25]. We therefore hypothesized that similar patterns of allelic differentiation between X and Y chromosomes would be observed across the populations if the sex chromosomes had been highly differentiated prior to postglacial recolonization. To gain insights into sex chromosome differentiation and population history, we also assessed the degree of genetic differentiation between and within the sex chromosomes across the populations using the sex-linked microsatellite marker.

## Materials and methods

### Samples

**Figure 1:**
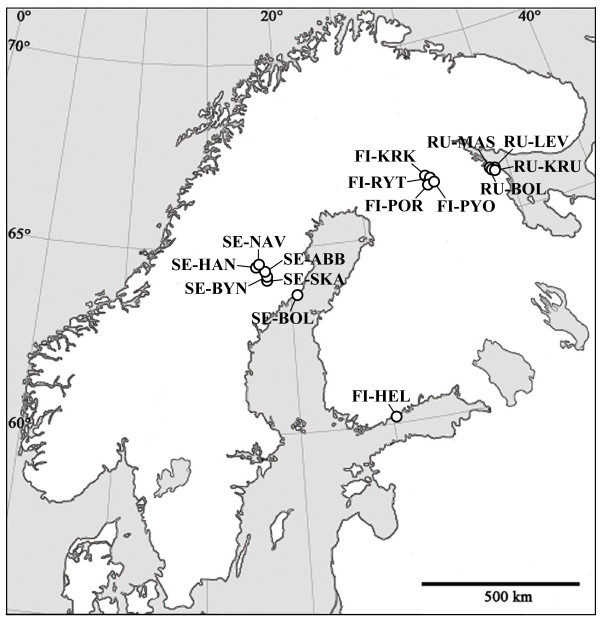
**Sampling locations of 15 study populations of Fennoscandian nine-spined sticklebacks**.

A total of 647 adult fish were collected with seine nets or minnow traps from 15 locations, comprising three coastal, two lake and 10 pond sites in Fennoscandia (Figure 1; Table 1). Phenotypic sex was initially determined by visual inspection (i.e. nuptial coloration of males and gravidity of females; 603 specimens) or by examining gonads (44 specimens) after the fish had been anesthetised with an overdose of MS-222 (tricane methanesulphonate). All procedures were performed under license from the Animal Experiment Board in Finland (ELLA; STH379A). Total DNA was extracted from fin clips using a silica-fine based purification method [26] following proteinase K digestion.

**Table 1:** Sampling sites and genetic variation at the microsatellite locus Stn19.

Site code	Site	Habitat type	Coordinates	*N*	*A*	*H*_E_	*F*_IS_
FI-KRK	Kirkasvetinenlampi	Pond	66°26' N, 29°08' E	55	2	0.391	-0.350
FI-RYT	Rytilampi	Pond	66°23' N, 29°19' E	46	2	0.104	-0.047
FI-PYO	Pyöreälampi	Pond	66°16' N, 29°26' E	62	2	0.324	-0.245
FI-POR	Iso-Porontima	Lake	66°13' N, 29°16' E	39	1	0.000	na
RU-LEV	Levin Navolok	Coastal	66°18' N, 33°24' E	34	2	0.163	-0.082
RU-MAS	Mashinnoje	Pond	66°18' N, 33°24' E	39	2	0.385	-0.333
RU-BOL	Bolotnoje	Pond	66°18' N, 33°24' E	35	3	0.393	-0.309
RU-KRU	Krugloje	Pond	66°18' N, 33°24' E	29	2	0.266	-0.167
SE-NAV	Lil-Navartjärn	Pond	64°34' N, 19°12' E	63	2	0.410	-0.393
SE-HAN	Hansmyrtjärn	Pond	64°33' N, 19°10' E	60	2	0.377	-0.326
SE-ABB	Abborrtjärn	Pond	64°29' N, 19°26' E	38	3	0.417	-0.326
SE-BYN	Bynästjärnen	Pond	64°27' N, 19°27' E	40	3	0.664	-0.129
SE-SKA	Vastre-Skavträsket	Lake	64°26' N, 19°27' E	30	1	0.000	na
SE-BOL	Bölesviken	Coastal	63°40' N, 20°13' E	39	4	0.639	-0.084
FI-HEL	Uutela	Coastal	60°12' N, 25°11' E	38	3	0.538	0.070

*N*, number of individuals; *A*, number of observed alleles; *H*_E_, expected heterozygosity; na, not applied.

### Genotyping

We surveyed genomic locations of the 11 three-spined stickleback microsatellite loci previously tested in nine-spined sticklebacks [27] by subjecting these flanking sequences to BLASTN searches against the three-spined stickleback genome [28]. The microsatellite locus Stn19 [29] was mapped to three-spined stickleback linkage group 12, which corresponds to sex chromosomes in the nine-spined stickleback [22,23]. We genotyped this marker in 647 individuals (399 phenotypic females and 248 phenotypic males) from the 15 populations listed in Table 1. The forward primer (5'-ACAGGCATGAATGACACTGG-3') was labelled with a fluorescent dye, and the 5'-end of the reverse primer (5'-GATGAGCACAACACCTGAGC-3') was modified with a GTTT-tail [30]. Polymerase chain reactions (PCRs) were carried out using the Qiagen Multiplex PCR Kit (Qiagen) in 10 μl reaction volumes containing 1× Qiagen Multiplex PCR Master Mix, 0.5× Q-Solution, 2 pmol of each primer and approx. 10-20 ng of template DNA. The reactions were performed by the following cycle: an initial activation step at 95°C for 15 min, followed by 30 s at 94°C, 90 s at 53°C and 60 s at 72°C for 30 cycles with a final extension at 60°C for 5 min. PCR products were visualized with a MegaBACE 1000 automated sequencer (Amersham Biosciences) and their sizes were determined with ET-ROX 550 size standard (Amersham Biosciences). Alleles were scored using Fragment Profiler 1.2 (Amersham Biosciences) with visual inspection and manual corrections of alleles.

### Data analyses

Expected heterozygosity and *F*_IS _were calculated according to population and sex using FSTAT 2.9.3 [31]. The significance of *F*_IS _was determined by 10 000 permutations. Sequential Bonferroni correction was applied to minimize type I errors [32]. To identify X and Y chromosomal alleles, genotypic frequencies and allele size distributions were compared between females and males. The degree of genetic differentiation in X and Y chromosomal alleles - both within and among populations - was evaluated using the (δμ)^2 ^distance, which is the squared difference in the number of microsatellite repeats [33]. This analysis was performed for the X and Y chromosome alleles identified in males, using Microsatellite Analyzer 4.05 [34]. Since two populations (FI-POR and SE-SKA; see also Results) were solely comprised of females (phenotypic and genotypic), these populations were excluded from this analysis. A UPGMA dendrogram was constructed from the (δμ)^2 ^distances using Populations 1.2 [35].

We note that the accuracy of phenotypic sexing was critical to our inference, and therefore specific care was taken to ensure that phenotypic sexes were correct. In our initial screen, 37 (5.7%) individuals showed a mismatch between phenotypic and genotypic (see below) sex. All the mismatches corresponded to individuals that had been sexed on the basis of visual criteria; re-examination of these individuals by gonadal inspection confirmed their true sex, which matched the genotypic sex perfectly.

## Results

Across the 15 populations, a total of five alleles were found at the locus Stn19 (Table 1). The number of observed alleles was three in females and four in males. In each population, one or two alleles were observed in females, whereas two to four alleles were detected in males (Table 2). No polymorphism was found in two populations (FI-POR and SE-SKA), from which only phenotypic females were available (Table 2). *F*_IS _values were nonsignificant for females in all populations (*P *> 0.05; Table 2). In contrast, all males were heterozygous at this locus, and accordingly, most populations exhibited significant negative *F*_IS _values in males (*P *< 0.05; Table 2).

**Table 2:** Genetic variation at the locus Stn19 in two sexes and X and Y chromosomes.

Site code	Female				Male				X	Y
				
	*N*	*A*	*H*_E_	*F*_IS_	*N*	*A*	*H*_E_	*F*_IS_	*A*	*A*
FI-KRK	26	1	0.000	na	29	2	0.500	-1.000*	1	1
FI-RYT	41	1	0.000	na	5	2	0.500	-1.000	1	1
FI-PYO	37	1	0.000	na	25	2	0.500	-1.000*	1	1
FI-POR	39	1	0.000	na	0	-	-	-	1	-
RU-LEV	28	1	0.000	na	6	2	0.500	-1.000	1	1
RU-MAS	19	1	0.000	na	20	2	0.500	-1.000*	1	1
RU-BOL	18	2	0.056	0.000	17	2	0.500	-1.000*	2	1
RU-KRU	20	1	0.000	na	9	2	0.500	-1.000	1	1
SE-NAV	27	1	0.000	na	36	2	0.500	-1.000*	1	1
SE-HAN	30	1	0.000	na	30	2	0.500	-1.000*	1	1
SE-ABB	19	2	0.102	-0.029	19	2	0.500	-1.000*	2	1
SE-BYN	19	2	0.515	0.080	21	3	0.624	-0.603*	2	1
SE-SKA	30	1	0.000	na	0	-	-	-	1	-
SE-BOL	19	2	0.494	0.254	20	4	0.609	-0.641*	2	2
FI-HEL	27	2	0.427	0.307	11	3	0.545	-0.833*	2	1

*N*, number of individuals; *A*, number of observed alleles; *H_E_*, expected heterozygosity; na, not applied. *P < 0.05.

**Figure 2:**
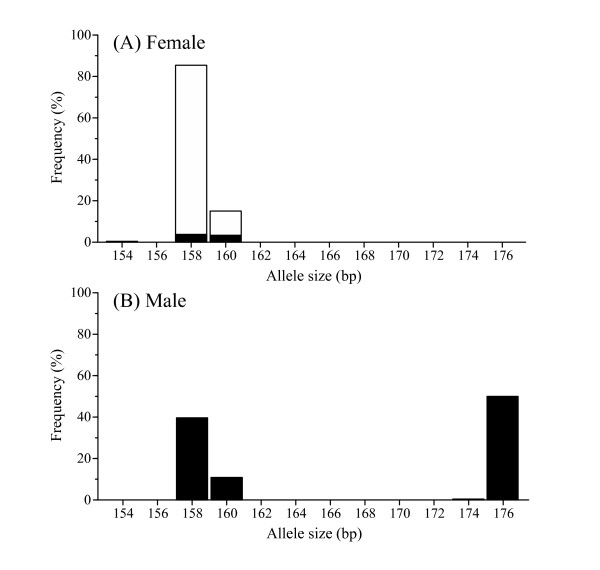
**Frequency distributions of alleles at the locus Stn19 in female (A) and male (B) nine-spined sticklebacks**. Open and solid bars indicate homozygous and heterozygous alleles, respectively.

Allele size distributions were clearly different between the sexes, and this pattern was consistent throughout the 15 populations (Figure 2). Only shorter (154, 158 and 160) alleles were identified in females, whereas both shorter and longer (174 and 176) alleles were detected in males (Figure 2). In fact, all males possessed one short and one long allele. Therefore, these longer alleles can be inferred to be located on the Y chromosome.

Assuming that the longer alleles are located on the Y chromosome, the number of alleles observed on this chromosome was one or two in each population (Table 2). Similarly, one or two alleles were observed in each population for the shorter alleles, which are presumed to be located on the X chromosome (Table 2). Polymorphism for Y chromosomal alleles was found only in one population (SE-BOL; Table 2). The number of alleles observed in each population was slightly but significantly larger in the alleles putatively located on the X chromosome than those on the Y chromosome (paired *t*-test, t_12 _= 2.31, *P *< 0.05). X chromosomal alleles were polymorphic in four out of seven populations from southern and central areas (60°12'-64°34' N), but these were monomorphic in most populations from northern areas (66°18'-66°26' N; Table 2). In the coastal populations, polymorphism was detected in the Baltic Sea populations (SE-BOL and FI-HEL) whereas no polymorphism was found in the White Sea population (RU-LEV; Table 2).

**Figure 3:**
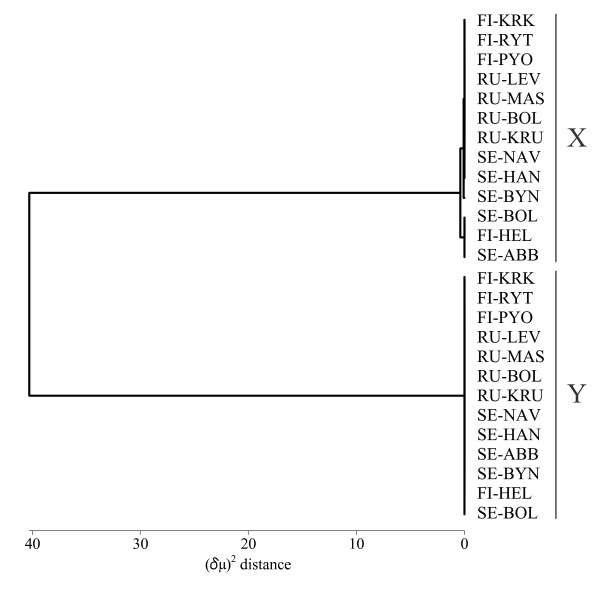
**UPGMA dendrogram based on (δμ)^2 ^distances of X and Y chromosomal alleles in 13 nine-spined stickleback populations**. X = X chromosomal alleles, Y = Y chromosomal alleles.

The (δμ)^2 ^distance was much larger between the X and Y chromosomal alleles than among alleles within each chromosome (Additional file 1). The UPGMA dendrogram constructed from the (δμ)^2 ^distances showed two major clusters corresponding to the X and Y chromosomal alleles across the populations (Figure 3). The (δμ)^2 ^distance between the X and Y chromosomal alleles was 64.0 to 81.0 in each population with a mean of 71.4 (SD = 6.9).

## Discussion

Our results demonstrated a clear and consistent difference in allele size distributions at the microsatellite locus Stn19 between sexes in a number of Fennoscandian populations. Males were distinguishable by the presence of distinct sex-specific alleles (174 and 176), whereas females were characterized by the absence of these alleles. Given the male heterogamy in this species [36], these male-specific alleles should be located on the Y chromosome. Since the observed pattern was consistent in a large number of individuals from various populations, allelic variation in this locus can be used as a diagnostic tool for sex identification in immature individuals, as well as in adults when phenotypic criteria are deemed to be unreliable. In our experience, phenotypic sexing by using external criteria is not possible at juvenile stages, and will sometimes lead to errors even in adult fish.

Despite our large sample size, we did not find any indication of recombination between the sex-determination locus and the Stn19 locus. Consistent patterns of sex-linked allelic differentiation were observed for other microsatellite loci located on the sex chromosomes independent of their physical location (T. Shikano, Y. Shimada & J. Merilä, unpublished). Therefore, distinct allelic divergence at the Stn19 locus between the sex chromosomes could be due to suppression of recombination between these chromosomes rather than due to tight physical linkage between the Stn19 locus and the sex-determination locus. Indeed, the sex chromosomes of nine-spined sticklebacks are cytogenetically distinguishable [36]. Thus, both molecular and cytogenetic characteristics indicate that sex chromosomes are highly differentiated from each other in this species. Yet, the fact that Stn19 and other sex-linked microsatellite loci (T. Shikano, Y. Shimada & J. Merilä, unpublished) maintain homologous amplification sites between the X and Y chromosomes suggests a high level of DNA similarity between these chromosomes. In the case of humans, where sex chromosomes initiated differentiation 240-320 million years ago [37], X and Y homologous microsatellite markers have been developed in limited homologous regions of these chromosomes [38-41].

Since northern Europe was covered by the continental ice sheet until approximately 12 000 years ago, nine-spined sticklebacks must have colonized Fennoscandia after the last glacial maximum. Based on an analysis of autosomal microsatellite markers, Fennoscandian populations are highly differentiated from each other perhaps due to founder effects following postglacial colonization [25]. Our study demonstrated that the level of genetic differentiation between the X and Y chromosomes was much larger than the interpopulation differentiation in the respective chromosomes. Because the patterns of allelic differentiation between the X and Y chromosomes were consistent in all populations, it is obvious that the sex chromosomes had been highly differentiated prior to the postglacial recolonization of Fennoscandia.

The usefulness of homologous microsatellite markers in X and Y chromosomes for the inferences of population history and sex-specific genetic structuring has been proposed in human studies [40,42-44]. Our results showed that the level of genetic variability in the X chromosome was lower in northern Fennoscandian populations as compared to those from central and southern Fennoscandia. This is consistent with a previous study that found a northward reduction in genetic variability at autosomal microsatellite loci [25]. In contrast, the level of genetic variability in the Y chromosome was consistently low in all populations. Similarly, mitochondrial DNA analyses revealed that one haplotype is widely distributed throughout the populations in the study area, suggesting that these populations share a common ancestry [25]. Hence, the low genetic variability of the paternal lineage supports the conclusion obtained from the maternal lineage. Further studies with more markers for the paternal lineage will be necessary to better evaluate this point, as well as to address sex-specific processes shaping population structure.

## Conclusions

In summary, we have demonstrated that the microsatellite locus Stn19 shows sex-specific allelic differences in a large number of Fennoscandian nine-spined stickleback populations. Thus, this marker can be used for the sex identification of immature individuals, as well as adults when visual criteria are deemed to be unreliable. The consistent patterns of allelic differentiation between these chromosomes across the populations suggest that the sex chromosomes of nine-spined sticklebacks had been highly differentiated prior to the postglacial recolonization of Fennoscandia.

## Competing interests

The authors declare that they have no competing interests.

## Authors' contributions

TS conceived the study, collected the molecular data, conducted the analyses and wrote the manuscript. GH collected the fish samples and recorded the phenotypic sex data. JM contributed to conceiving and writing of the manuscript. All authors read and approved the final manuscript.

## Supplementary Material

Additional file 1 **Matrix of (δμ)^2 ^distances of X and Y chromosomal alleles within and between 13 nine-spined stickleback populations**. Click here for file
