# Supplementary material for: Molecular sexing and population genetic inference using a sex-linked microsatellite marker in the nine-spined stickleback (Pungitius pungitius)
Source: BMC Res Notes. 2011 Apr 12;4:119. doi: 10.1186/1756-0500-4-119 (PMC3094369; doi:10.1186/1756-0500-4-119)
Supplement: Additional file 1 — Matrix of (δμ)2 distances of X and Y chromosomal alleles within and between 13 nine-spined stickleback populations. [file 1756-0500-4-119-S1.PDF]

Additional file 1: Matrix of $(\delta\mu)^2$ distances of X and Y chromosomal alleles within and between 13 nine-spined stickleback populations.

	X chromosome													Y chromosome												
	FI-KRK	FI-RYT	FI-PYO	RU-LEV	RU-MAS	RU-BOL	RU-KRU	SE-NAV	SE-HAN	SE-ABB	SE-BYN	SE-BOL	FI-HEL	FI-KRK	FI-RYT	FI-PYO	RU-LEV	RU-MAS	RU-BOL	RU-KRU	SE-NAV	SE-HAN	SE-ABB	SE-BYN	SE-BOL	FI-HEL
X chromosome																										
FI-KRK																										
FI-RYT	0.0																									
FI-PYO	0.0	0.0																								
RU-LEV	0.0	0.0	0.0																							
RU-MAS	0.0	0.0	0.0	0.0																						
RU-BOL	0.0	0.0	0.0	0.0	0.0																					
RU-KRU	0.0	0.0	0.0	0.0	0.0	0.0																				
SE-NAV	0.0	0.0	0.0	0.0	0.0	0.0	0.0																			
SE-HAN	0.0	0.0	0.0	0.0	0.0	0.0	0.0	0.0																		
SE-ABB	1.0	1.0	1.0	1.0	1.0	1.0	1.0	1.0	1.0																	
SE-BYN	0.1	0.1	0.1	0.1	0.1	0.1	0.1	0.1	0.1	0.4																
SE-BOL	0.6	0.6	0.6	0.6	0.6	0.6	0.6	0.6	0.6	0.0	0.2															
FI-HEL	0.8	0.8	0.8	0.8	0.8	0.8	0.8	0.8	0.8	0.0	0.3	0.0														
Y chromosome																										
FI-KRK	81.0	81.0	81.0	81.0	81.0	81.0	81.0	81.0	81.0	64.0	74.3	67.2	65.5													
FI-RYT	81.0	81.0	81.0	81.0	81.0	81.0	81.0	81.0	81.0	64.0	74.3	67.2	65.5	0.0												
FI-PYO	81.0	81.0	81.0	81.0	81.0	81.0	81.0	81.0	81.0	64.0	74.3	67.2	65.5	0.0	0.0											
RU-LEV	81.0	81.0	81.0	81.0	81.0	81.0	81.0	81.0	81.0	64.0	74.3	67.2	65.5	0.0	0.0	0.0										
RU-MAS	81.0	81.0	81.0	81.0	81.0	81.0	81.0	81.0	81.0	64.0	74.3	67.2	65.5	0.0	0.0	0.0	0.0									
RU-BOL	81.0	81.0	81.0	81.0	81.0	81.0	81.0	81.0	81.0	64.0	74.3	67.2	65.5	0.0	0.0	0.0	0.0	0.0								
RU-KRU	81.0	81.0	81.0	81.0	81.0	81.0	81.0	81.0	81.0	64.0	74.3	67.2	65.5	0.0	0.0	0.0	0.0	0.0	0.0							
SE-NAV	81.0	81.0	81.0	81.0	81.0	81.0	81.0	81.0	81.0	64.0	74.3	67.2	65.5	0.0	0.0	0.0	0.0	0.0	0.0	0.0						
SE-HAN	81.0	81.0	81.0	81.0	81.0	81.0	81.0	81.0	81.0	64.0	74.3	67.2	65.5	0.0	0.0	0.0	0.0	0.0	0.0	0.0	0.0					
SE-ABB	81.0	81.0	81.0	81.0	81.0	81.0	81.0	81.0	81.0	64.0	74.3	67.2	65.5	0.0	0.0	0.0	0.0	0.0	0.0	0.0	0.0	0.0				
SE-BYN	81.0	81.0	81.0	81.0	81.0	81.0	81.0	81.0	81.0	64.0	74.3	67.2	65.5	0.0	0.0	0.0	0.0	0.0	0.0	0.0	0.0	0.0	0.0			
SE-BOL	80.1	80.1	80.1	80.1	80.1	80.1	80.1	80.1	80.1	63.2	73.4	66.4	64.7	0.0	0.0	0.0	0.0	0.0	0.0	0.0	0.0	0.0	0.0	0.0		
FI-HEL	81.0	81.0	81.0	81.0	81.0	81.0	81.0	81.0	81.0	64.0	74.3	67.2	65.5	0.0	0.0	0.0	0.0	0.0	0.0	0.0	0.0	0.0	0.0	0.0	0.0	
